# Supplementary material for: Experience in the adoption of Research Electronic Data Capture (REDCap) as a tool for medical research in Tanzania
Source: medRxiv. 2025 Oct 28:2025.10.25.25338769. Preprint. [Version 1] doi: 10.1101/2025.10.25.25338769 (PMC12636652; doi:10.1101/2025.10.25.25338769)
Supplement: 1 [file NIHPP2025.10.25.25338769V1-supplement-1.pdf]

medRxiv preprint doi: https://doi.org/10.1101/2025.10.25.25338769; this version posted October 28, 2025. The copyright holder for this preprint (which was not certified by peer review) is the author/funder, who has granted medRxiv a license to display the preprint in perpetuity.
It is made available under a CC-BY 4.0 International license .

Supplementary Materials

Experience in the adoption of Research Electronic Data Capture (REDCap) as a tool for medical research in Tanzania

Running title: REDCap for medical research in Tanzania

Raphael Zozimus Sangeda[1*], Upendo Masamu[2], Daniel Kandonga[2], Fredrick Mbuya[2], Liberata Mwita[1,2], Frank Makundi[2], Josephine Mgaya[2], Agnes J. Jonathan[2,], Elisha Osati[2,3], Bruno P. Mmbando[2,4], Siana Nkya[2,5], Emmanuel Balandya[2,6], Julie Makani[2,7]

[1]Department of Pharmaceutical Microbiology, School of Pharmacy, Muhimbili University of Health and Allied Sciences, Dar es Salaam, Tanzania.

[2]Muhimbili Sickle Cell Program, Department of Hematology and Blood Transfusion, Muhimbili University of Health and Allied Sciences, Dar es Salaam, Tanzania.

[3]Muhimbili National Hospital, Dar es Salaam, Tanzania

[4]National Institute for Medical Research, Tanga Center, Tanga, Tanzania

[5]Department of Biochemistry, Muhimbili University of Health and Allied Sciences (MUHAS), Dar-es-Salaam, Tanzania.

[6]Department of Physiology, Muhimbili University of Health and Allied Sciences, Dar es Salaam, Tanzania.

[7]Department of Hematology and Blood Transfusion, Muhimbili University of Health and Allied Sciences (MUHAS), Dar-es-Salaam, Tanzania.

Contact and ORCID

RZS[*]; Corresponding author: sangeda@gmail.com, +255757582643, 0000-0002-6574-5308

medRxiv preprint doi: https://doi.org/10.1101/2025.10.25.25338769; this version posted October 28, 2025. The copyright holder for this preprint (which was not certified by peer review) is the author/funder, who has granted medRxiv a license to display the preprint in perpetuity.
It is made available under a CC-BY 4.0 International license .

Supplementary Figure

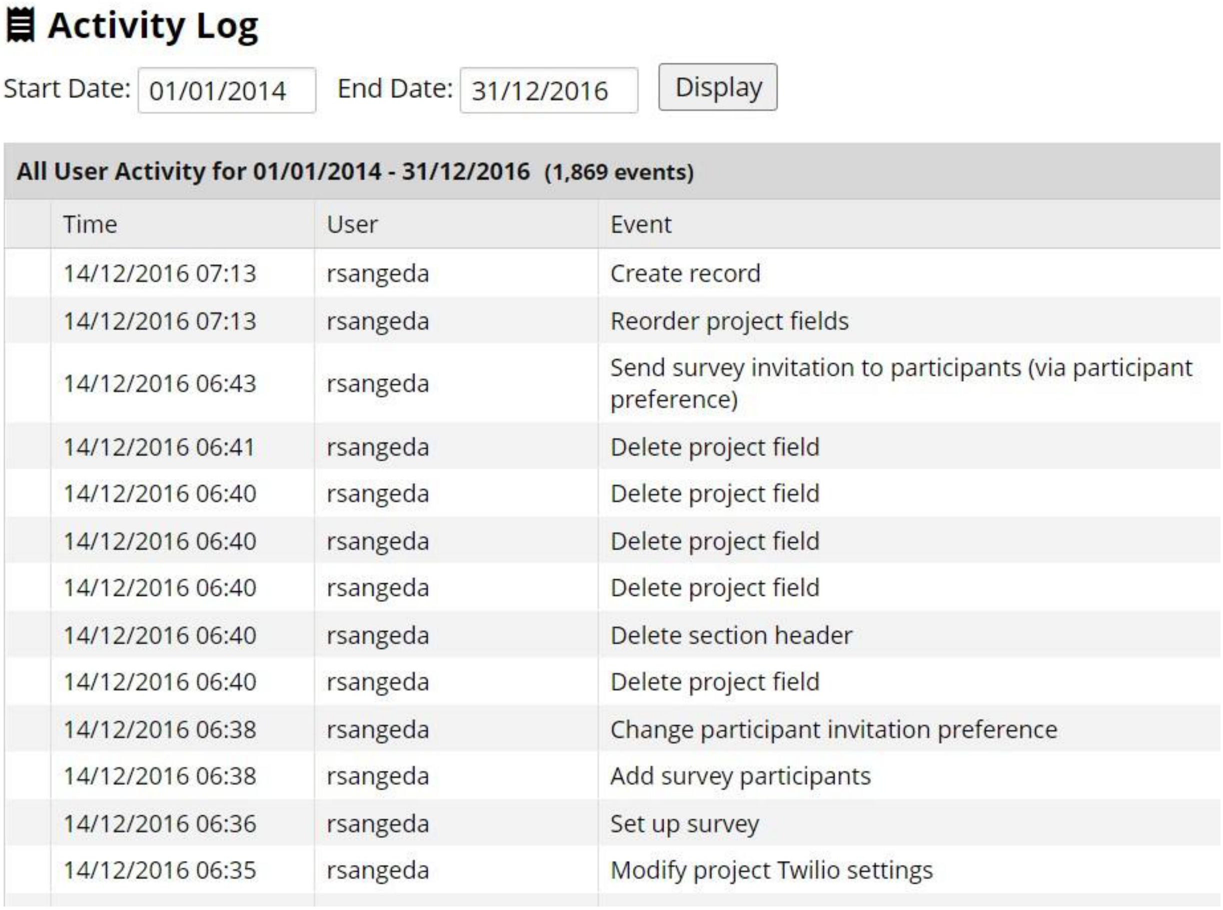

Activity Log

Start Date: 01/01/2014 End Date: 31/12/2016 Display

All User Activity for 01/01/2014 - 31/12/2016 (1,869 events)

Time	User	Event
14/12/2016 07:13	rsangeda	Create record
14/12/2016 07:13	rsangeda	Reorder project fields
14/12/2016 06:43	rsangeda	Send survey invitation to participants (via participant preference)
14/12/2016 06:41	rsangeda	Delete project field
14/12/2016 06:40	rsangeda	Delete project field
14/12/2016 06:40	rsangeda	Delete project field
14/12/2016 06:40	rsangeda	Delete project field
14/12/2016 06:40	rsangeda	Delete section header
14/12/2016 06:40	rsangeda	Delete project field
14/12/2016 06:38	rsangeda	Change participant invitation preference
14/12/2016 06:38	rsangeda	Add survey participants
14/12/2016 06:36	rsangeda	Set up survey
14/12/2016 06:35	rsangeda	Modify project Twilio settings

Supplementary Figure 1: Early activities evidence of logged events in REDCap - truncated to show a few events from the REDCap server events log
